# Continuous pericapsular nerve group block for postoperative pain management in total hip arthroplasty: report of two cases

**DOI:** 10.1186/s40981-021-00423-1

**Published:** 2021-03-07

**Authors:** Takashi Fujino, Masahiko Odo, Hisako Okada, Shinji Takahashi, Toshihiro Kikuchi

**Affiliations:** 1 Department of Anesthesiology and Pain Medicine, Juntendo Nerima University Hospital, 3-1-10 Takanodai, Nerima-ku, Tokyo, 177-8521 Japan; 2 Department of Anesthesiology and Pain Medicine, Juntendo University Urayasu Hospital, 2-1-1 Tomioka, Urayasu-shi, Chiba, 279-0021, Japan

**Keywords:** Pericapsular nerve group (PENG) block, Continuous peripheral nerve block, Total hip arthroplasty, Peripheral nerve block

## Abstract

**Background:**

Total hip arthroplasty (THA) is one of the surgical procedures associated with severe postoperative pain. Appropriate postoperative pain management is effective for promoting early ambulation and reducing the length of hospital stay. Effects of conventional pain management strategies, such as femoral nerve block and fascia iliaca block, are inadequate in some cases.

**Case presentation:**

THA was planned for 2 patients with osteoarthritis. In addition to general anesthesia, continuous pericapsular nerve group (PENG) block and lateral femoral cutaneous nerve (LFCN) block were performed for postoperative pain management. Numerical rating scale (NRS) scores measured at rest and upon movement were low at 2, 12, 24, and 48 h postoperatively, suggesting that the treatments were effective for managing postoperative pain. The Bromage score at postoperative days (POD) 1 and 2 was 0.

**Conclusion:**

Continuous PENG block and LFCN block were effective for postoperative pain management in patients who underwent THA. PENG block did not cause postoperative motor blockade.

## Background

Total hip arthroplasty (THA) is associated with significant postoperative pain [1]. Opioids are typically used for postoperative pain management in patients undergoing THA; however, intravenous patient-controlled analgesia (IV-PCA) with opioids can delay ambulation due to side effects [2]. Although used frequently, the effects of conventional pain management strategies, such as femoral nerve block and fascia iliaca block, are inadequate in some cases [3]. Furthermore, postoperative motor blockade can prevent early ambulation [4].

Pericapsular nerve group (PENG) block was reported as the first method that targets the nerves that supply the hip capsule [5]. Subsequently, several studies reported that it was more effective for managing postoperative pain associated with THA than conventional peripheral nerve blocks [6]. However, a single-injection PENG block is only effective for a limited time. In THA, there is little evidence supporting the effectiveness of continuous PENG block for postoperative pain management. Here, we report two cases of successful postoperative pain control for THA using anesthesia with continuous PENG block and lateral femoral cutaneous nerve (LFCN) block. Consent was received from the patients, and the study was approved by the institutional research ethics board (S19-60).

## Case presentation

### Anesthesia application

After establishing standard monitoring, general anesthesia was induced with propofol, rocuronium, and remifentanil. Supraglottic device (LMA ProsealTM) was uneventfully performed. Subsequently, ultrasound-guided LFCN block and PENG block were performed. General anesthesia was maintained by the target-controlled infusion (TCI) of propofol, in addition to remifentanil and fentanyl. Acetaminophen (1000 mg) was administered 15 min before the end of the surgery. For postoperative pain management, continuous PENG block was performed with 0.2% ropivacaine at 6 mL/h for 48 h.

### Pain evaluation

Postoperative pain was assessed at rest and upon movement at 2, 12, 24, and 48 h postoperatively using the numerical rating scale (NRS) score. On postoperative days (POD) 1 and 2, the level of motor blockade was assessed using the Bromage score (0, free movement; 1, inability to raise the extended leg and can bend the knee; 2, inability to bend the knee and can flex the ankle; 3, no movement).

### Block intervention

**Fig. 1:**
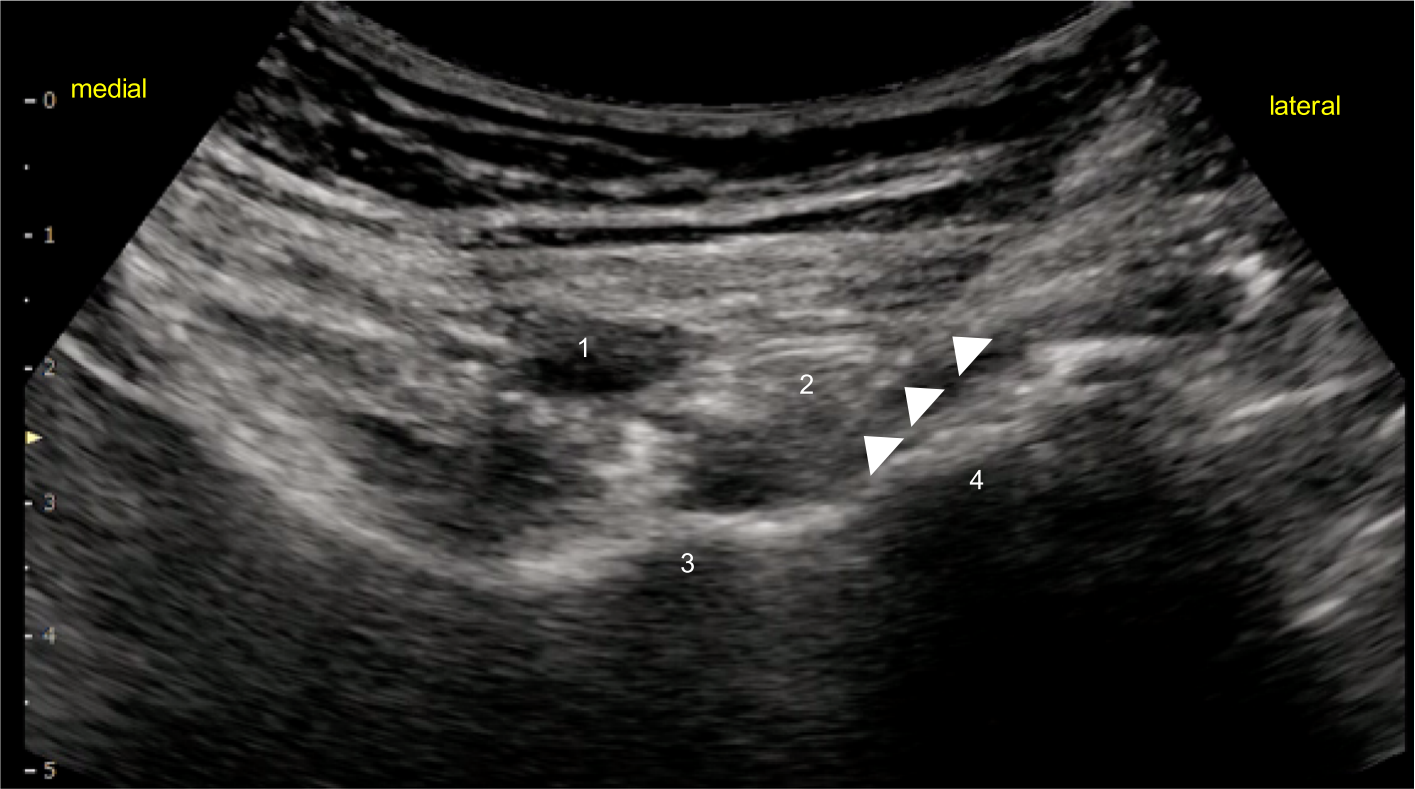
Ultrasound images showing sonoanatomy while performing pericapsular nerve group block. White arrowheads shows the block needle. 1 = femoral artery; 2 = psoas tendon; 3 = iliopubic eminence; 4 = anteroinferior iliac spine

**Fig. 2:**
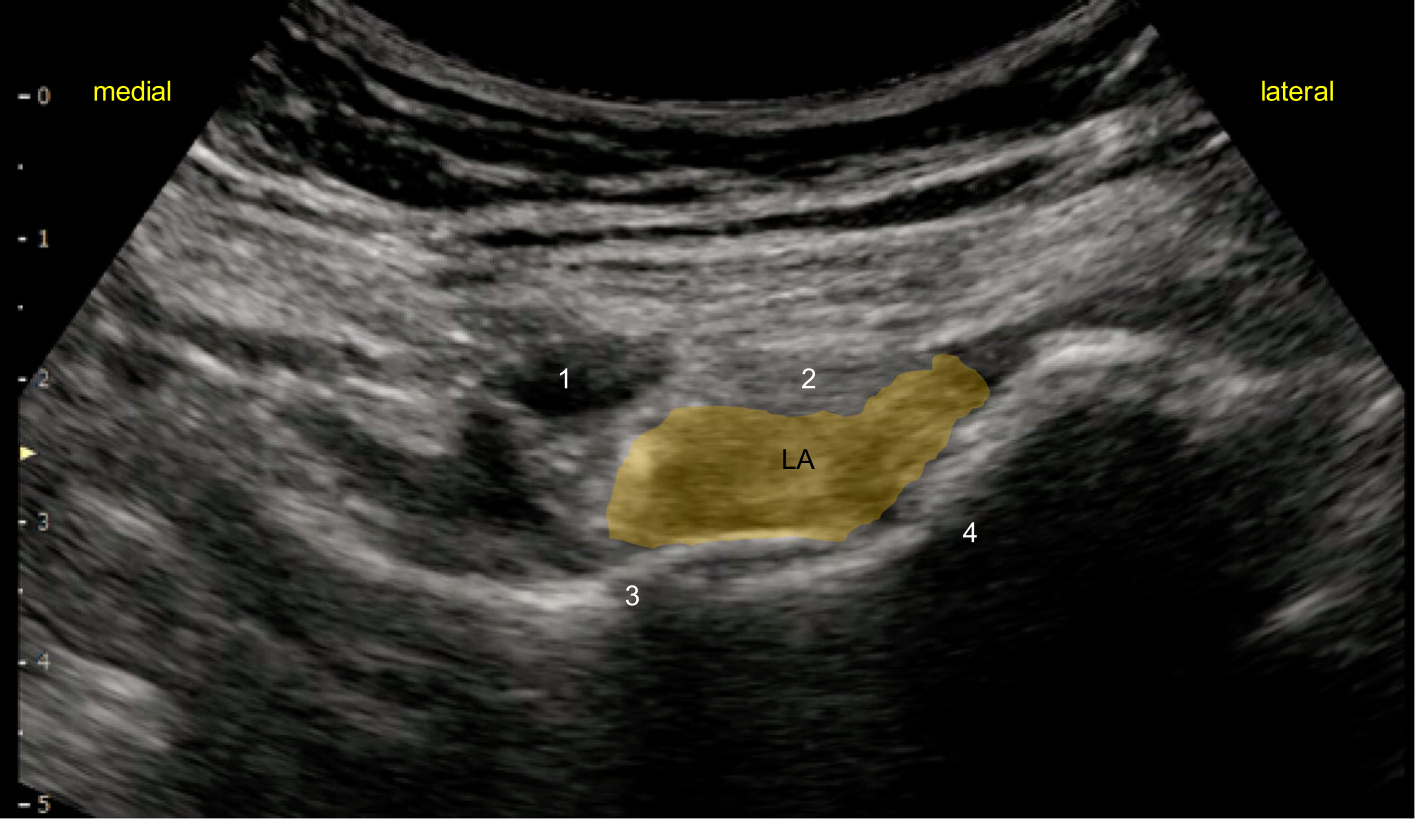
The local anesthetic spread following injection. LA, local anesthetic

After induction of general anesthesia, LFCN block was performed using a high-frequency (4–14 Hz) linear probe (SONIMAGE MX1, KONICA MINOLTA JAPAN, Tokyo, Japan) and a 22-gauge 80-mm needle (Contiplex® Tuohy Ultra, B BRAUN, Tokyo, Japan). A 5–10 mL of 0.375% ropivacaine was injected around the LFCN. Subsequently, PENG block was performed. Patients were placed in the supine position, and the anterior inferior iliac spine was identified by placing a low-frequency (2–5 Hz) convex probe slightly towards the head and parallel to the groin. The probe was rotated 45° counterclockwise along the inferior pubic ramus to identify the iliopubic eminence (Fig. 1). An 18-gauge 100-mm Tuohy needle was inserted in parallel from the outside into the iliopubic eminence. The needle was inserted deep into the iliopsoas tendon, and 20 mL of 0.5% ropivacaine was injected after ensuring that there was no blood drawn back from the site of injection (Fig. 2). From the same site, a continuous catheter was inserted 4 cm from the tip of the needle without resistance. The location of the tip of the catheter was confirmed by injecting air, and the catheter was left deep in the iliopsoas tendon. The catheter was fixed to the puncture site inside the anterior inferior iliac spine, cranial to the inguinal ligament, to prevent interference with the surgical field.

### Case 1

The patient was a 71-year-old woman (body weight, 49 kg; height, 148 cm) with a history of hypertension. THA was performed due to osteoarthritis of the hip using a posterolateral approach. General anesthesia was maintained by propofol (TCI, 2.3–3.3 μg/mL) and remifentanil (0.15 μg/kg/h) with stable hemodynamics. A total of 200 μg of fentanyl was used during surgery. Postoperatively, the patient developed mild pain upon movement in the outer side of the thighs but did not require additional pain management. The patient was able to start using a walker and increase loading on the affected side from POD1, with a Bromage score of 0 (Table 1).

**Table 1:** The demographics, NRS score after surgery, Bromage scale, and number of analgesic use

Case	Sex	Age,y	Side	NRS at rest				NRS at movement				Bromage scale		Number of analgesic use
				2h	12h	24h	48h	2h	12h	24h	48h	POD1	POD2	
1	F	71	R	0	0	0	0	0	2	2	0	0	0	0
2	F	72	L	0	0	0	0	0	3	3	2	0	0	2

*NRS* Numerical rating scale

### Case 2

The patient was a 72-year-old woman (body weight, 45 kg; height, 141 cm) with a history of dyslipidemia. THA was performed due to osteoarthritis of the hip using a posterolateral approach. General anesthesia was maintained with propofol (TCI, 1.6–2.5 μg/mL) and remifentanil (0.1 μg/kg/h) with stable hemodynamics. A total of 50 μg of fentanyl was used during surgery. In the recovery room, the patient had no complaints of pain. She subsequently developed pain upon movement in the outer side of the thighs 8 h after the surgery and was administered celecoxib as a rescue analgesic. At 48 h after surgery, the pain was well-managed with low NRS scores both at rest and upon movement (Table 1). The patient was able to start using a wheelchair and walk on POD1 with a Bromage score of 0.

## Discussion

We described 2 cases in which continuous PENG block and LFCN block are effective for postoperative pain management in patients undergoing THA. PENG block did not cause postoperative motor blockade.

Continuous PENG block is effective for postoperative pain management in patients undergoing THA. A recent cadaveric study demonstrated that sensory nerves are most abundant in the anterior hip capsule, which is supplied by the superior branch of the femoral nerve, obturator nerve, and accessory obturator nerve [7]. Studies also suggest that the accessory obturator nerve plays a particularly important role in innervation of hip capsules. The superior branch of the femoral nerve and accessory obturator nerve are found between the anterior inferior iliac spine and iliopubic eminence to supply the hip capsule. Although PENG block targets these nerves that supply the hip capsule, femoral nerve block and fascia iliaca block do not target them. Of note, a previous study examined the injectate distribution with fascia iliaca block using magnetic resonance imaging (MRI) and confirmed that the drug did not reach the obturator nerve [8]. Furthermore, a cadaveric study used methylene blue and demonstrated that the dye penetrated primarily into the anterior hip capsule when administered with PENG block [9]. Collectively, consistent with a previous study [5], PENG block may be more effective as an analgesic than conventional peripheral nerve block for patients undergoing hip surgery. However, in these studies, a single-injection PENG block was performed, which has a limited duration of action. We demonstrated that continuous PENG block was effective for maintaining low NRS scores soon after surgery and for up to 48 h after the surgery, thus enabling patients to start using a wheelchair and walk on POD1.

In our cases, PENG block did not cause postoperative motor blockade. As PENG block specifically targets the sensory branch to the hip that branches out from the main trunk of the femoral nerve, it may be possible to preserve the motor branch of the femoral nerve [5]. Although several studies demonstrated that femoral nerve block and fascia iliaca block cause postoperative motor blockade [10], both of our patients had a Bromage score of 0, and neither experienced knee buckling when getting up from the bed. Thus, PENG block may be effective for promoting early ambulation as it can preserve the motor branch of the femoral nerves. However, Yu et al. reported that 2 of over 100 patients who underwent a single-injection PENG block developed motor blockade [11]. They demonstrated that for cases in which blockade is technically challenging to perform (i.e., in obese patients), PENG can block the motor branch of the femoral nerves if the drug (1) has spread to the superficial layer of the iliopsoas muscle, (2) has mostly spread within the iliopsoas tendon, or (3) is injected into the iliopsoas muscle due to failed needle insertion into the iliopsoas fascia. However, due to the limited evidence, additional studies are needed to fully elucidate the motor-sparing effects of PENG block. In the present cases, the needle was successfully inserted deep into the iliopsoas tendon to perform PENG block such that the drug was distributed equally deep into the iliopsoas tendon (Fig. 2).

Supra-inguinal fascia iliaca compartment block (SI-FICB) enables a more extensive spread of a local anesthetic to the parietal region than conventional fascia iliaca compartment block, resulting in the consistent block of three target nerves of the lumbar plexus, i.e., femoral, obturator, and lateral femoral cutaneous nerves. Therefore, many reports have been published on its use for hip surgery [12]. However, this method remains controversial regarding obturator nerve block. Furthermore, the combination of SI-FICB with obturator nerve block in hip surgery improved postoperative analgesic effects compared with SI-FICB alone, suggesting that SI-FICB cannot ensure obturator nerve block [13]. On the other hand, the use of 30 mL of a local anesthetic in the PENG block can concurrently block the obturator, femoral, and lateral femoral cutaneous nerves, suggesting the same effects as lumbar plexus block [14]. PENG block can be performed in a supine position and is technically easier and safer than lumbar plexus block. However, caution is needed to prevent motor nerve block during the use of high doses of local anesthetics.

Singh et al. used PENG block by a single-shot technique with a 10-mL bolus dose of 0.25% bupivacaine followed by continuous infusion of 0.25% bupivacaine at the rate of 5 ml/h [15]. Unexpected motor nerve block was avoided by minimizing the diffusion of the drug solution to the surface. They reported satisfactory pain relief. However, as the PENG block is a “plane” block that does not directly target the nerves, sufficient diffusion of local anesthetic is essential. The novelty of the present case is that motor nerve block was avoided even with the bolus dose of 20 mL by moving the needle to administer local anesthetic at the appropriate site.

The wound in the present case was located on the lateral side of the proximal thigh, which is perceived mainly in the lateral femoral cutaneous nerve area. To improve pain control at the early stage of surgery, lateral femoral cutaneous nerve block in addition to PENG block may be required. There is no report on concurrent lateral femoral cutaneous nerve block with 20 mL of local anesthetic in the PENG block. As high doses increase the risk of motor nerve block, lateral femoral cutaneous nerve block was combined with continuous PENG block, resulting in satisfactory analgesic effects without motor nerve block.

As Tran et al. explained in their paper [9], injection of 20 mL of methylene blue extensively stained both the entire anterior hip joint capsule and the bursal space between the iliopsoas and anterior hip joint capsule, and the spread pattern provides support that a “true” pericapsular block. Though we did not confirm the spread pattern posterior to the articular capsule, this may explain the effective pain relief in our cases. Further study is needed to determine the optimal volume for this block as Tran mentioned.

However, the more reliable explanation for good pain relief with PENG block even in posterolateral approach might be the distribution of the nerve fibers. The anterior capsule has been found to have the highest density of sensory nerve fibers in the hip joint [16, 17]. A recent review of histological studies examining the innervation of the hip joint capsular complex shows that the posterior aspect of the capsule has a much lower density of sensory nerves than the anterior aspect of the capsule [18]. Furthermore, it has been previously shown that nociceptive hip joint capsule fibers primarily innervate the anterior aspect of the capsule, while the nerve fibers innervating the posterior aspect of the capsule are mainly mechanoreceptors [19]. These findings suggest that blocking the nerves innervating the anterior capsule is the most important factor in achieving good analgesia and that PENG block is a reasonable technique for postoperative analgesia in THA.

In conclusion, our findings suggest that anesthesia with continuous PENG block and LFCN block is an effective option for postoperative pain management in patients undergoing THA, and PENG block did not cause postoperative motor blockade.

## Data Availability

Please contact the corresponding author for data requests.

## Abbreviations

THA: Total hip arthroplasty
PENG: Pericapsular nerve group
LFCN: Lateral femoral cutaneous nerve
NRS: Numeric rating scale
IV-PCA: Patient-controlled analgesia
TCI: Target-controlled infusion
MRI: Magnetic resonance imaging
